# Did It Change Your Mind? Neural Substrates of Purchase Intention Change and Product Information

**DOI:** 10.3389/fnins.2022.871353

**Published:** 2022-05-09

**Authors:** Hesun Erin Kim, Joon Hee Kwon, Jae-Jin Kim

**Affiliations:** ^1^Institute of Behavioral Science in Medicine, Yonsei University College of Medicine, Seoul, South Korea; ^2^Department of Psychiatry, Yonsei University College of Medicine, Seoul, South Korea

**Keywords:** price, customer rating, purchase intention, frontopolar cortex, salience processing

## Abstract

Price and customer ratings are perhaps the two most important pieces of information consumers rely on when shopping online. This study aimed to elucidate the neural mechanism by which the introduction of these two types of information influences the purchase intention of potential consumers for hedonic products. Participants performed a lip-care product shopping task during functional magnetic resonance imaging, in which they re-disclosed purchase intentions referring to the information of price or rating provided about the products that they had previously disclosed their purchase intentions without any information. Data from 38 young female participants were analyzed to identify the underlying neural regions associated with the intention change and product information. The bilateral frontopolar cortex, bilateral dorsal anterior cingulate cortex (dACC), and left insula activated higher for the unchanged than changed intention condition. The right dACC and bilateral insula also activated more toward the price than the rating condition, whereas the medial prefrontal cortex and bilateral temporoparietal junction responded in the opposite direction. These results seem to reflect the shift to exploratory decision-making strategies and increased salience in maintaining purchase intentions despite referring to provided information and to highlight the involvement of social cognition-related regions in reference to customer ratings rather than price.

## Introduction

Advances in technology and the outbreak of coronavirus 2019 (COVID-19) have aided the meteoric explosion of the e-commerce marketplace. A survey by McKinsey & Company suggests an average of 30% growth in online purchases due to the onset of the pandemic (Charm et al., 2020). Similar to the decision-making process of offline shopping, even in online ones, if consumers have a need, they search for information, evaluate alternatives, make a purchase decision, and assess post-purchase satisfaction. Compared to traditional offline shopping, online shopping boasts greater convenience and efficiency as it allows consumers to browse and shop products with more options beyond store hours from the comfort of their home (Wang et al., 2005).

Despite these advantages, uncertainty and lack of trust remain intrinsic problems of online shopping because consumers cannot physically assess products. In order to mitigate this issue and encourage sales, retailers provide a wide range of information about their products on online platforms, and consumers rely heavily on that information to make purchasing decisions. In fact, it is understood that besides the perceived security concerns and interactive experience with the website, the amount of information available in a product has a significant impact on consumer behavior (Ballantine, 2005). Details such as brand, descriptions, images, price, reviews, and ratings are important cues that consumers seek in their decision-making process.

The use of neuroimaging techniques, particularly functional magnetic resonance imaging (fMRI), has been under the spotlight as a powerful tool in understanding the consumption behavior in recent decades (Lim, 2018a). The consensus is that rigorous empirical investigations and available resources for new researchers coming into this multidisciplinary field of consumer neuroscience are still lacking and rather heterogeneous (Lee et al., 2018). In an effort to expand the transdisciplinary field of consumer neuroscience or neuromarketing, recent publications offer a comprehensive overview of the different applications of neuroscientific methods, research designs, and possible ethical issues (Lim, 2018b). In addition, information on how data should be managed and processed using actual data specific to business research has widened the scope and boundaries of neuromarketing studies (Lim, 2018b; Robaina-Calderin and Martin-Santana, 2021).

Different modes of visualization in online shopping are an excellent way to mimic a brick-and-mortar experience and reduce the perceived risk. For example, online retailers have started to offer virtual try-on technology, which allows users to directly interact with products by zooming and rotating them (Kim and Forsythe, 2008; Jai et al., 2014). Interestingly, several neuroimaging studies have investigated the neural substrates of product presentation, where these sensory-enabling presentations engage the superior parietal lobule associated with mental imagery and the ventral striatum related to reward processing (Jai et al., 2014; Kim et al., 2021). These findings have confirmed the significance of product presentation and elucidated the neural engagements while the product is being evaluated.

Price information is also one of the most important factors that influence consumer behavior in an online setting and has been extensively researched (Kim et al., 2012; Beneke and Carter, 2015). The perception of price is an extremely complicated cue because it is not only a monetary sacrifice in exchange for a product or service but also a delivery of product quality (Lichtenstein et al., 1993). Perceived price points can have both positive and negative effects on purchase intentions, such that a higher price may indicate higher quality, but may discourage consumers if perceived as excessive (Dodds et al., 1991).

A previous neuroimaging study reported the role of the medial prefrontal cortex (mPFC) and insula in the prediction of consumer behavior in response to price (Knutson et al., 2007). In this study, mPFC activity increased when consumers saw a lower-than-expected price and predicted purchase behavior, whereas insula activity was increased when the price was perceived too high. The order in which price information is exposed to consumers is also believed to be crucial in shaping product preferences. Another neuroimaging study reported differences in the activation of the mPFC, suggesting the primacy effect in the consumer decision-making process (Karmarkar et al., 2015). Interestingly, in this study, mPFC activity increased for purchased over non-purchased products in the product primacy condition, but not in the price primacy condition, and striatal activity increased for purchased products regardless of the primacy conditions, suggesting that seeing the price before the product promoted consumers to consider its monetary worth, whereas seeing the product before price encouraged them to focus on its desirability. Taken together, the way a product is presented influences not only purchasing behavior but also neural responses, particularly the reward process.

Online stores include customer ratings, which are related to the positive effect of word-of-mouth on purchasing behavior (Chintagunta et al., 2010; Ye et al., 2011; Anderson and Magruder, 2012; Kim et al., 2012), and are considered to be a major factor influencing purchasing decisions (Bughin et al., 2010; Floyd et al., 2014). Extensive research has demonstrated that product reviews and consumer's purchase intentions have a positive relationship (Chevalier and Mayzlin, 2006; Park et al., 2007). Although written and descriptive product reviews are important for shaping purchase intentions, previous studies show that average customer ratings are highly influential as well, especially among younger shoppers (Hong and Park, 2012; von Helversen et al., 2018).

When the influence of others' opinions was studied using fMRI, it was found that conflict with group opinion activated the rostral cingulate zone and deactivated the ventral striatum, both of which are known to compute prediction errors (Klucharev et al., 2009), suggesting that such neural changes may lead to the realization for the need to conform and trigger behavioral adjustment. Several other neuroimaging studies have also shown conforming to group opinion that activates the ventral striatum and medial orbitofrontal cortex (mOFC), suggesting that accepting social norm is rewarding (Campbell-Meiklejohn et al., 2010; Zaki et al., 2011; Cascio et al., 2015). Furthermore, social influences in the decision-making process may be important in this issue, and brain regions that deserve special attention are the temporoparietal junction (TPJ) and posterior superior temporal sulcus (pSTS), which are regarded as the key to understanding the mental states of others or mentalization (Saxe and Kanwisher, 2003). When actual salespeople were recruited as participants, it was found that TPJ activity was associated with the ability to read their customer's minds (Dietvorst et al., 2009). Social influence of own decisions was instantaneously reflected in the TPJ, and its activity was actually modulated by opposing opinions of others (Zhang and Gläscher, 2020). In addition, the mPFC has been associated with the social decision-making process. For example, using products and their ratings from an online retail site, mPFC activity was found to be modulated by the reliability of social information and confidence of own judgment in a Bayesian fashion (De Martino et al., 2017).

There is an intricate interaction between the price and customer ratings of a retail product. Ratings are more influential for high-end products than low-end products because higher prices allow consumers to utilize information like product reviews to mitigate the perceived risk (Maslowska et al., 2017). Additionally, lowering the price of products with low customer ratings has been shown to alleviate its adverse effects and increase purchasing behavior (Kuo and Nakhata, 2016).

The consumer decision process is certainly influenced by the type of product involved. Cosmetics are considered hedonic goods, a broad category of products that are purchased for enjoyment, emotion regulation, and self-enhancement, and carry social meaning, as opposed to those for problem-solving (utilitarian goods) (Dhar and Wertenbroch, 2000; Ajitha and Sivakumar, 2017). Consumers are more sensitive to others' opinions and price points in hedonic goods than in utilitarian goods when in doubt about the products (Parry and Kawakami, 2015; Ajitha and Sivakumar, 2017). Although the literature on consumer behavior largely highlights the importance of customer ratings and prices on the consumer decision-making process, there is a substantial gap between behavioral and neuroscientific evidence.

Because of the involvement of the simultaneous processing of such a large amount of information, consumer decision-making is expected to engage multiple cognitive control mechanisms. Previous studies point out the dorsolateral prefrontal cortex (dlPFC), dorsal anterior cingulate cortex (dACC), and caudate as key structures associated with these cognitive control processes (Egner and Hirsch, 2005; Botvinick, 2007; Stelzel et al., 2010). The highly connected nature of the dlPFC seems to contribute to various cognitive processes such as decision strategy, inhibition, and reasoning (Clark and Manes, 2004). The dACC has been shown to activate toward conflicting options and facilitate decision-making by assessing the decision-making strategy generated by the dlPFC (Wallis, 2007). The caudate is another important region as several studies have shown its involvement in adaptive decision-making (Tricomi and Lempert, 2015; Doi et al., 2020). Social cognition is another component that plays a role in the consumer decision-making process, as shopping is a highly social activity, especially when it involves others' product opinions. The literature generally reports the mPFC, TPJ, and pSTS as cardinal brain areas involved in social cognition (Gallagher and Frith, 2003; Kramer et al., 2010; Olson et al., 2013). This involvement of multiple functions suggests that neural regions respond differently throughout the course of consumer decision-making.

The purpose of this study was to elucidate the neural mechanism by which the introduction of product information influences the purchase intention of potential consumers for hedonic goods and to identify the neural regions associated with the level of intention change. The investigation also aimed to probe how these functions are rendered during evaluation and choice phases of the decision-making process. We expected that cognitive processes related to decision-making, including cost-benefit calculation and action control, and social cognition would respond distinctively depending on the purchase intention change and product information. Accordingly, we hypothesized that the prefrontal regions, dACC, and caudate, which are important in making decision strategies and other cognitive processes, would be involved in information-related changes in purchase intention during the evaluation phase, whereas the temporal areas and TPJ, structures known for their role in social cognition, would respond in favor of customer ratings during the choice phase. In addition, we hypothesized that increases in purchase intention would be correlated with the magnitudes of ventral striatum activity and mOFC activity, as they are critical nodes in the reward circuit.

## Materials and Methods

### Participants

A total of 42 healthy young female volunteers were recruited *via* online advertisement. Exclusion criteria included left-handedness, pregnancy, and neurological or psychiatric diseases. All participants were provided informed written consent before the study; the study was approved by the Institutional Review Board of Yonsei University Severance Hospital and carried out in accordance with the Declaration of Helsinki. Data from four participants were excluded from the analysis due to excessive missing trials (>15%) or skewed distribution of intention change scores, and thus, data from the remaining 38 participants (age, 23.6 ± 2.0; education years, 16.2 ± 1.6) were used for the analysis.

### Experimental Procedure

**Figure 1:**
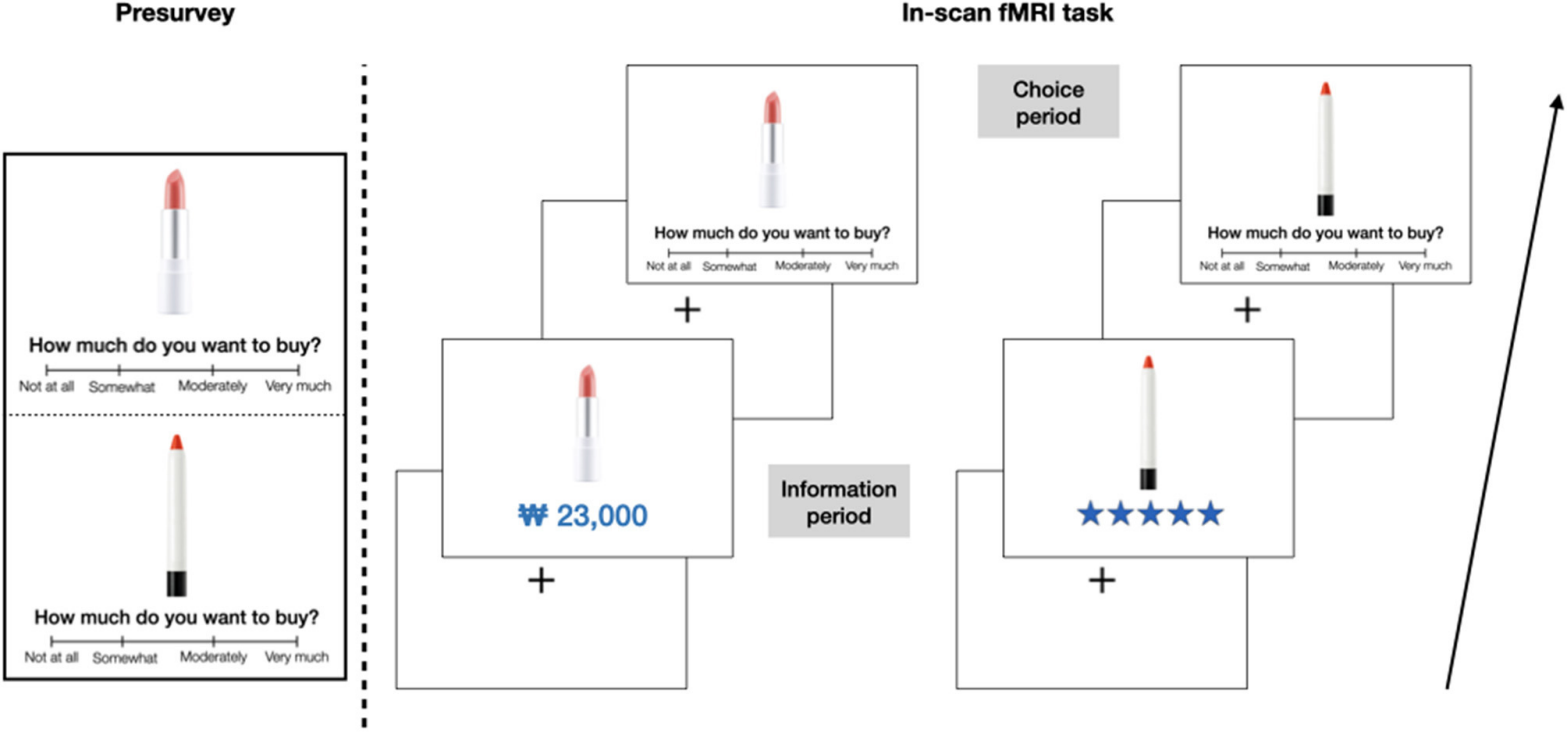
Experimental design. Prior to the fMRI task, an online presurvey was conducted, where participants were asked to indicate their level of purchase intention on a 4-point Likert scale for each lip-care product. In the fMRI task, the same products were presented either with price or star rating information for 2 s, and then, participants disclosed the level of purchase intention on the same scale once more.

Participants performed a lip-care product shopping task (Figure 1) during fMRI. The task included a total of 84 lip-care images collected from various online cosmetic websites. All images were cropped to show only the content and body without the lid and were presented on a white background. All brand marks were erased to eliminate the branding effect. In a presurvey conducted prior to the fMRI session, participants responded to the question “How much do you want to buy?” on a 4-point Likert scale (0: “not at all”, 1: “somewhat”, 2: “moderately”, 3: “very much”) to indicate the level of purchase intention for each product. No information about price or rating was disclosed to them at this point.

In the lip-care product shopping task, 84 items were randomly assigned to either price or rating condition so that price and rating could influence the purchasing decision. The price information for each product was randomly generated, ranging from 5,000 Korean Won (KRW; approximately US$4.50) to 35,000 KRW (approximately US$31.50). The average price was about 17,300 KRW (approximately US$15.70). Customer ratings were represented in five-star images. The rating information was randomly assigned to each of 42 products, ranging from 0.5 to 5 stars, with an average of 2.9 stars. The information was placed beneath the product and presented in blue.

The task was composed of two runs, with each run having 42 trials and lasting approximately 7 min 10 s. One lip-care product was presented per trial, where price or rating information was presented for 2 s (information period). After jittering an average of 2 s, participants were asked to answer the same question as given in the presurvey in a time of 3 s on a 4-point Likert scale (choice period). Intervals between trials were jittered for an average of 3 s.

### Behavioral Data Analysis

For each product, the change in purchase intention was calculated by subtracting the purchase intention score at the presurvey from the purchase intention score during the fMRI session. A score difference of zero was considered no change (“unchanged”) and a non-zero score difference was deemed as being changed in the level of intention (“changed”). First, to check the validity of dividing the trials as such, the proportions of trials were compared using a one-sample binomial test at 0.5 test proportion. Once determined to be appropriate, change scores were compared between the two product information factors (price and rating) using the chi-square test. Behavioral data were analyzed using SPSS 25.0 (SPSS, Inc., Chicago, IL).

### Imaging Data Acquisition

All functional scanning was performed on 3.0 Tesla MRI scanner (Ingena 3.0T CX, Philips Healthcare, Best, NL) with a 32-channel head coil. For each participant, echo-planar imaging scans were acquired with the following parameters: field of view = 224, repetition time = 2,000 ms, echo time = 30 ms, flip angle = 90°, number of acquisitions = 215, number of slices = 31, slice thickness = 3 mm with 1 mm interstitial gap, and matrix size = 80 × 80. A high-resolution T1-weighted anatomical scan was also obtained from each participant using a 3D gradient echo (field of view = 224, number of slices = 220, slice thickness = 1 mm, matrix size = 224 × 224) after the functional scan.

### Imaging Data Preprocessing and Statistical Analysis

The first five scans were discarded for magnetic field stabilization. The rest of the images were preprocessed and analyzed using SPM12 (https://www.fil.ion.ucl.ac.uk/spm/). Functional data were realigned for head motion correction and corrected for slice-timing. Head movement artifacts were assessed in individual subjects to confirm that the maximum head motion in each axis was <3 mm. Individual anatomical image was coregistered to mean functional images coregistered on the individual anatomical image, spatially normalized to Montreal Neurological Institute (MNI) template space, and then smoothed with a Gaussian kernel of 6-mm full-width at half-maximum.

Once preprocessed, general linear model (GLM) was performed in the first-level analysis. Two types of analyses were performed, namely, categorical and parametric, for the intention change given respective product information. To identify the underlying neural regions associated with the intention change and product information, four main regressors (i.e., changed-price, unchanged-price, changed-rating, and unchanged-rating) were created at the information period for each run. Each trial was modeled at the onset time of the information period with the duration of 2 s. Another GLM was conducted with the same four main regressors of interest modeled at the respective onset time of the choice period with the duration of the reaction time (RT) for each run (Grinband et al., 2008). Additional six nuisance regressors were included as regressors-of-no-interest, and a high-pass filter was applied at 128 Hz to correct for low-frequency drift and physiological noise.

At the second level, the resulting four contrast images modeled for each participant were entered into the flexible factorial model for the main effects and interaction effects between the two factors in each of the information and choice periods. *Post-hoc* analysis was performed to identify the direction of differences by extracting parameter estimates of each significant cluster with a radius of 5 mm sphere using MarsBaR 0.44.

The second type of analysis, parametric analysis, was performed to investigate the neural regions modulated by the level of intention change given the price or rating information. The two product information main regressors were entered, and then, the change scores of each valid trial were included as parametric regressors to estimate neural responses for each period. Onset, duration, convolution, and six nuisance regressors-of-no-interest were set the same as those in GLM. Positive and negative relationships were tested separately between neural signals and change scores for each information condition. The resulting contrasts were submitted to one-sample *t*-tests. All statistical inferences were set at a threshold of family-wise error (FWE) corrected *P*_*FWE*_ < 0.05 at the cluster level with a cluster-defining threshold of uncorrected *p* < 0.001.

## Results

### Behavioral Data

We first assessed the proportions of changed and unchanged trials using a one-sample binomial test. The overall proportions were 48.9% (SD = 0.098) for changed condition and 51.1% (SD = 0.098) for unchanged condition and did not significantly differ between the two conditions (*P* > 0.05). Factoring in price and rating information, the chi-square test revealed no difference between purchase intention change and product information types ($X^{2}$ =1.03, *P* > 0.05).

### Imaging Data

#### Effects of Intention Change During the Evaluation of Product Information

**Figure 2:**
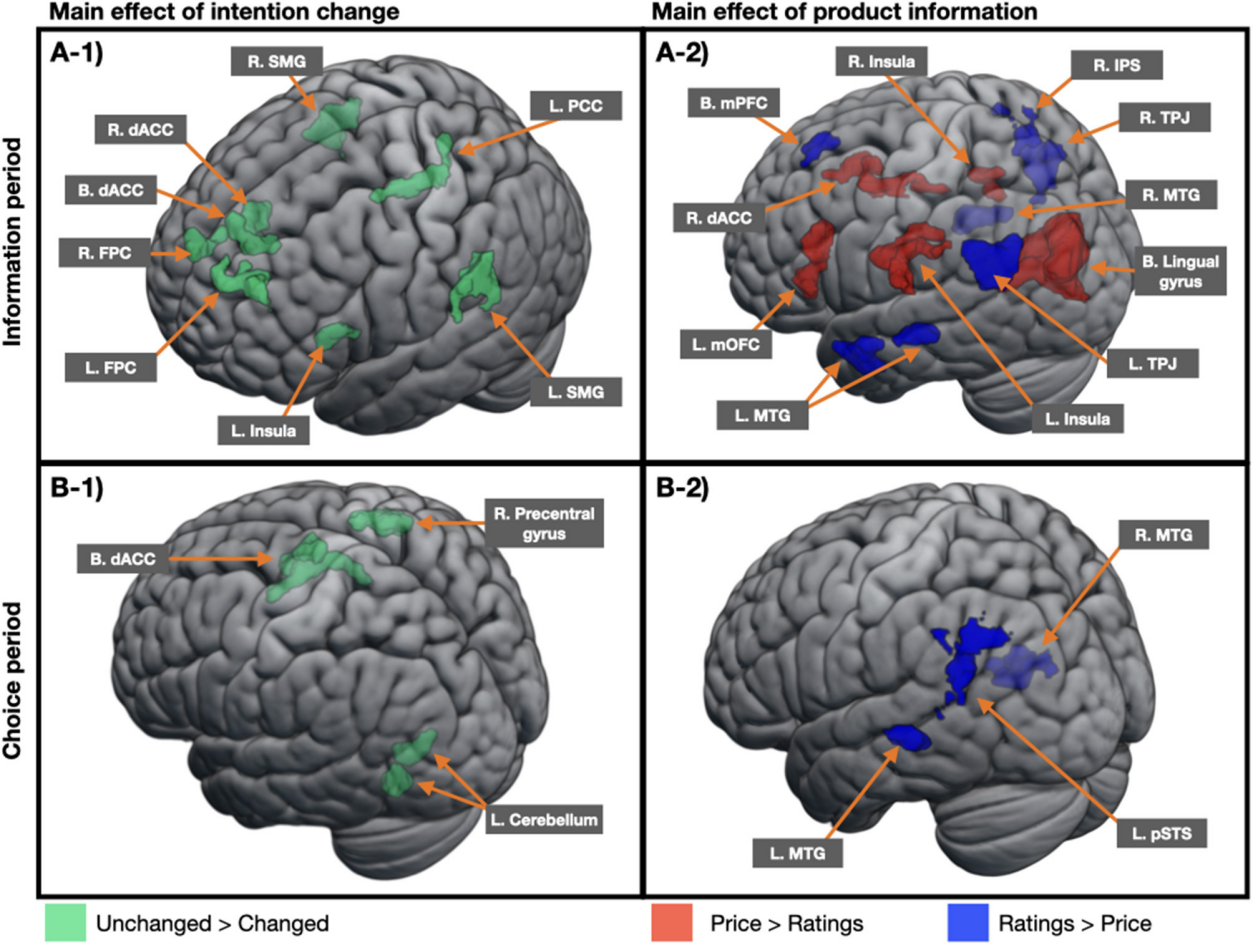
An illustration of neural regions showing the main effects of intention change and product information in the categorical analysis. All regions showing the main effect of intention change were more activated in the unchanged condition than in the changed condition at both the information period **(A-1)** and choice period **(B-1)**. **(B)** The regions showing the main effect of product information were mixed with those that responded more to the price condition and those that responded more to the rating condition at the information period **(A-2)**, but were more activated in the rating condition than in the price condition at the choice period **(B-2)**. B, bilateral; L, left; R, right; FPC, frontopolar cortex; dACC, dorsal anterior cingulate cortex; PCC, posterior cingulate cortex; SMG, supramarginal gyrus; mOFC, medial orbitofrontal cortex; mPFC, medial prefrontal cortex; MTG, middle temporal gyrus; TPJ, temporoparietal junction; IPS, intraparietal sulcus; pSTS, posterior superior temporal sulcus.

Table 1 presents the neural regions showing the main effects of intention change and product information and their interaction effects at the information period. The main effect of intention change was identified in the bilateral frontopolar cortex (FPC), bilateral dACC, left posterior cingulate cortex, bilateral supramarginal gyrus, and left insula. As shown in Figure 2A**-1**, *post-hoc* analysis indicated that those regions were more activated in the unchanged condition than in the changed condition, whereas no region was more activated in the changed condition than in the unchanged condition. The main effect of product information was observed in multiple brain regions. *Post-hoc* analysis indicated that among these regions, the left mOFC, right dACC, bilateral lingual gyrus, and bilateral insula responded more to the price condition than to the rating condition, whereas the bilateral mPFC, bilateral middle temporal gyrus, bilateral TPJ, and right intraparietal sulcus responded more to the rating condition than to the price condition (Figure 2B**-1**). However, no significant interaction effects were found.

**Table 1:** Main and interaction effects of intention change and product information at the information period.

				**MNI coordinates**			
**Region**	**HEM**	**Cluster size**	**F**	**X**	**Y**	**Z**	***Post hoc***
**Main effect of intention change**							
FPC	L	201	16.57	−12	54	22	Unchanged > Changed
FPC	R	148	21.77	22	48	16	Unchanged > Changed
dACC	B	136	19.57	0	28	24	Unchanged > Changed
dACC	R	118	18.57	16	30	24	Unchanged > Changed
PCC	L	196	19.63	−14	−32	44	Unchanged > Changed
SMG	L	308	25.61	−60	−38	28	Unchanged > Changed
SMG	R	200	22.44	60	−40	34	Unchanged > Changed
Insula	L	112	21.35	−22	12	−14	Unchanged > Changed
**Main effect of product information**							
mOFC	L	320	35.30	−22	34	−6	Price > Ratings
dACC	R	338	21.06	16	26	30	Price > Ratings
Lingual gyrus	B	1,988	41.34	8	−58	2	Price > Ratings
Insula	L	489	24.60	−36	−2	16	Price > Ratings
Insula	R	119	20.19	34	−32	24	Price > Ratings
mPFC	B	113	19.30	6	34	48	Ratings > Price
MTG	L	285	31.11	−52	0	−20	Ratings > Price
MTG	L	155	29.71	−60	−26	−10	Ratings > Price
MTG	R	181	38.25	62	−18	−10	Ratings > Price
TPJ	L	580	31.83	−50	−60	28	Ratings > Price
TPJ	R	385	33.64	56	−52	28	Ratings > Price
IPS	R	109	16.06	36	−46	44	Ratings > Price
**Interaction effect**							
None							

*HEM, hemisphere; MNI, Montreal Neurologic Institute; B, bilateral; R, right; L, left; FPC, frontopolar cortex; dACC, dorsal anterior cingulate cortex; PCC, posterior cingulate cortex; SMG, supramarginal gyrus; mOFC, medial orbitofrontal cortex; mPFC, medial prefrontal cortex; MTG, middle temporal gyrus; TPJ, temporoparietal junction; IPS, intraparietal sulcus*.

#### Effects of Product Information During the Choice Process

Table 2 presents the neural regions showing the main effects of intention change and product information and their interaction effects at the choice period. The main effect of intention change was identified in the bilateral dACC, right precentral gyrus, and left cerebellum. As shown in Figure 2A**-2**, *post-hoc* analysis indicated that those regions were more activated in the unchanged condition than in the changed condition, whereas no region was more activated in the changed condition than in the unchanged condition. The main effect of product information was observed in the left pSTS and bilateral middle temporal gyrus. As shown in Figure 2B**-2**, *post-hoc* analysis indicated that those regions were more activated in the rating condition than in the price condition, whereas no region was more activated in the price condition than in the rating condition. However, no significant interaction effects were found.

**Table 2:** Main and interaction effects of intention change and product information at the choice period.

				**MNI coordinates**			
**Region**	**HEM**	**Cluster size**	**F**	**X**	**Y**	**Z**	***Post hoc***
**Main effect of intention change**							
dACC	B	441	25.36	8	6	42	Unchanged > Changed
Precentral gyrus	R	233	18.93	28	−24	60	Unchanged > Changed
Cerebellum	L	148	22.74	−12	−60	−14	Unchanged > Changed
Cerebellum	L	119	20.42	−26	−62	−20	Unchanged > Changed
**Main effect of product information**							
pSTS	L	315	23.38	−46	−54	26	Ratings > Price
MTG	L	122	28.55	−62	−36	−2	Ratings > Price
MTG	R	280	24.76	62	−26	−2	Ratings > Price
**Interaction effect**							
None							

*HEM, hemisphere; MNI, Montreal Neurologic Institute; B, bilateral; R, right; L, left; dACC, dorsal anterior cingulate cortex; pSTS, posterior superior temporal sulcus; MTG, middle temporal gyrus*.

#### Parametric Modulation of Intention Change

**Figure 3:**
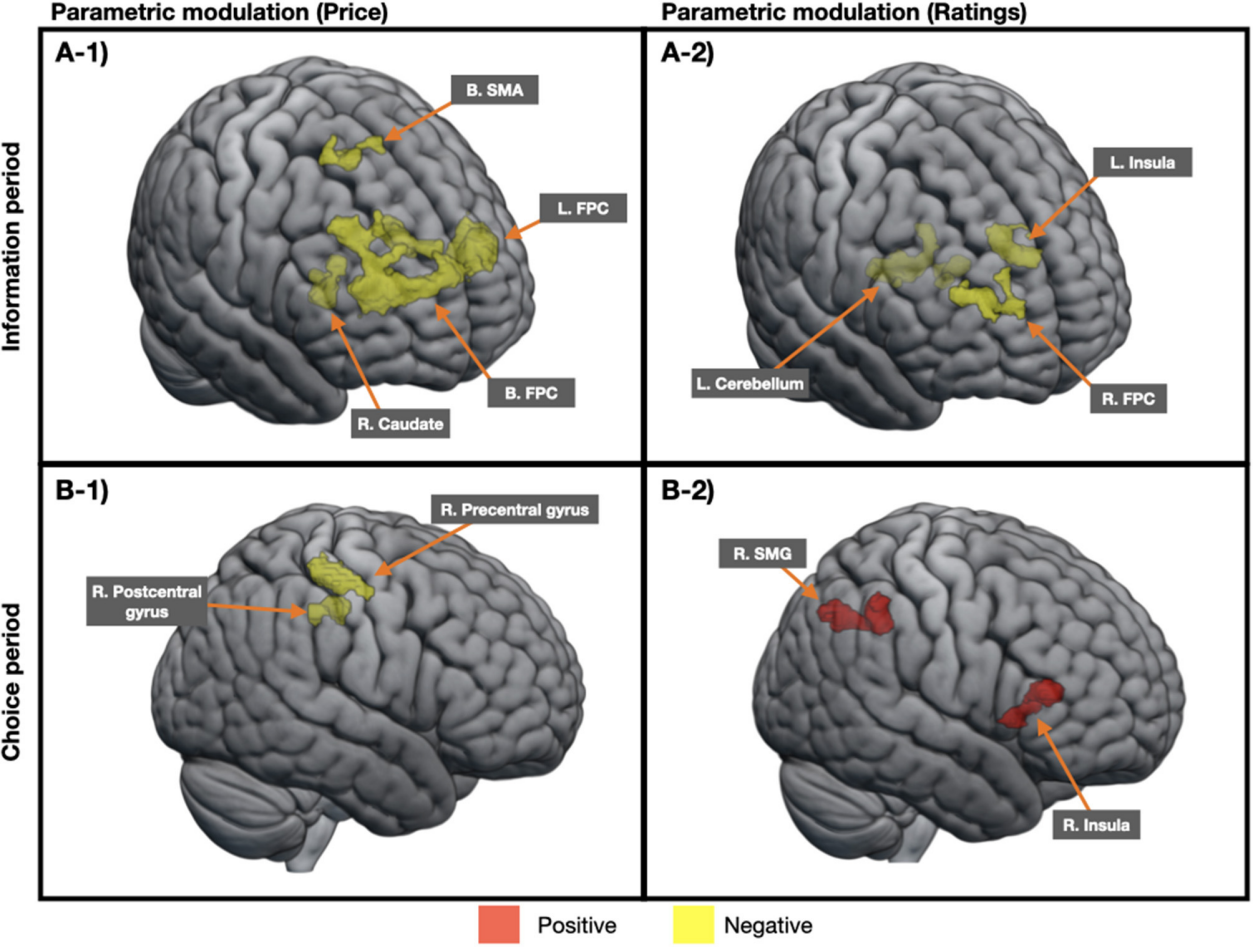
An illustration of regions associated with the intention change scores in parametric modulation analysis. At the information period, regions showing the negative parametric effect were observed in both the price condition **(A-1)** and rating condition **(A-2)**. On the contrary, at the choice period, regional activities were negatively modulated in the price condition **(B-1)** and positively modulated in the rating condition **(B-2)**. B, bilateral; L, left; R, right; FPC, frontopolar cortex; SMA, supplementary motor area; SMG, supramarginal gyrus.

Table 3 presents results from parametric modulation analysis of intention change. At the information period, the intention change score negatively modulated the activities of the bilateral FPC, bilateral supplementary motor area, and right caudate for price information (Figure 3A**-1**) and also negatively modulated the activities of the right FPC, left insula, and left cerebellum for rating information (Figure 3A**-2**). However, no regional activity was positively modulated by the intention change score at this period. At the choice period, the intention change score negatively modulated the activities of the right precentral and postcentral gyri for price information (Figure 3B**-1**), whereas it positively modulated the activities of the right supramarginal gyrus and right insula for rating information (Figure 3B**-2**).

**Table 3:** Results from parametric modulation of intention change.

				**MNI coordinates**			
**Region**	**HEM**	**Cluster size**	**T**	**X**	**Y**	**Z**	**Direction**
**Price**							
**Information period**							
FPC	L	492	−5.33	−24	46	24	Negative
FPC	B	1,060	−5.12	2	58	20	Negative
SMA	B	154	−4.45	−4	8	64	Negative
Caudate	R	207	−4.14	18	8	14	Negative
Choice period							
Precentral gyrus	R	213	−5.06	38	−24	60	Negative
Postcentral gyrus	R	142	−4.60	34	−24	44	Negative
**Ratings**							
**Information period**							
FPC	R	199	−4.13	14	54	16	Negative
Insula	L	177	−4.61	−22	22	12	Negative
Cerebellum	L	402	−4.97	−22	−66	−20	Negative
Choice period							
SMG	R	391	4.54	46	−40	42	Positive
Insula	R	202	4.69	38	16	2	Positive

*HEM, hemisphere; MNI, Montreal Neurologic Institute; B, bilateral; R, right; L, left; FPC, frontopolar cortex; SMA, supplementary motor area; SMG, supramarginal gyrus*.

## Discussion

This study provided evidence that price and customer ratings may influence purchase intentions for hedonic products, and this influence may be represented differently depending on the stage of the decision-making process. Unlike the original hypothesis, the change scores did not differ between the price and rating conditions, and no region showed significant interaction effects between intention change and product information. As expected, however, cognitive control structures responded differently to products with changed and unchanged purchase intentions during the information period, and social cognition structures responded differently to rating and price information during the choice period. Notably, these responses were stronger for the unchanged than changed condition and for rating than price information. Overall, analyses identified the prominence of strategy, social cognition, and salience processing in the consumer decision-making process.

The most characteristic region found in the main effect of intention change at the information period was the FPC. This region has been regarded as a vital component in the higher order cognition, such as reasoning, goal monitoring, strategy, and goal-directed behavior in decision-making (Koechlin et al., 1999; Ramnani and Owen, 2004; Daw et al., 2006; Kim et al., 2011; Mansouri et al., 2017). Engagement in exploration strategies for goal-directed behavior is essential to achieve an optimal decision-making outcome in complex and dynamic situations, like shopping. Exploration strategies refer to gathering different information, estimating the cost and benefit values of available options, and redistributing resources accordingly. Although we initially expected the dlPFC to be involved because of its relevance to cognitive control and decision strategy, the present data suggest a greater reliance of exploration strategies over exploitation strategies (Mansouri et al., 2017). Previous studies have consistently indicated the unique role of the FPC in the operation of exploration strategies (Daw et al., 2006; Laureiro-Martinez et al., 2014; Mansouri et al., 2017). There is also a report that the activation of the FPC is related to efficient decisions based on cognitive flexibility in an exploitation-exploration decision-making task demanding profit maximization (Laureiro-Martinez et al., 2014).

Based on these roles, FPC activation for unchanged over changed purchase intention demonstrated in our data may convey greater simultaneous assessment of goals, including current and alternative, computation of decision values, and reallocation of cognitive resources to achieve the best outcome when purchase intentions did not change. In addition, parametric modulation analysis showed that FPC activity during the information period was negatively modulated by the purchase intention change scores for both the price and rating conditions. In other words, FPC activity increased as the intention to purchase decreased regardless of price or ratings. Given that the FPC is a key player in the exploratory decision-making, such modulatory effect seen in the data may also communicate that the shift from exploitation strategies to information-driven exploration strategies underlies the process in which the introduction to price or rating curbs one's desire to purchase. Our results suggest that these cognitive processes may occur more even when evaluating products rather than making choices, which seems logical in that it requires strategizing before making a choice.

Other regions showing the main effect of intention change at the information period included the dACC and insula. Given that these two regions are critical members of the salience network that aids decision-making (Uddin et al., 2017), the preferential activation may imply that intention-unchanged beauty products are more salient than intention-changed products. A previous study using social acceptance and rejection tasks also elucidated that activations of these areas were related to salience processing rather than negative experiences (Perini et al., 2018). In particular, the dACC was also more activated in the unchanged than changed condition at the choice period, but the insula was not. Decades of research suggest that the dACC is highly interconnected with other regions in the brain and contributes to a variety of functions, such as cognitive control, calculation of costs and benefits of actions, and behavioral adjustment and salience processing (Alexander and Brown, 2011; Uddin et al., 2017; Yee et al., 2021). Furthermore, this region is thought to calculate the expected values of control that are sensitive to reward and punishment (Shenhav et al., 2013; Lake et al., 2019) and contribute to the cognitive control process by integrating the incentive values (Yee et al., 2021). Taken together, the cognitive control process of the dACC including these various functions seems to have a strong effect on the maintenance of purchase intentions despite the interference of provided information.

The two salience-related regions, namely, dACC and insula, also showed the main effect of product information, reacting more strongly in the price condition than in the rating condition. Previous evidence associates the dACC with cognitive functions and the insula with affective functions within the framework of salience processing (Critchley et al., 2004; Menon and Uddin, 2010; Shenhav et al., 2013; Gogolla, 2017). Therefore, increased activity toward price information seen in the main effect of product information may indicate a greater saliency of price, which prompts cognitive control by calculating the expected values of control and integrating the subjective motivational values. In addition, the mOFC was also found to show a preference for price information over rating information, which is understandable in that the region is often known to be related to subjective valuation and preference in many decision-making studies (O'Doherty, 2011; Westbrook et al., 2019).

On the contrary, the mPFC and TPJ were more activated in the rating condition than in the price condition at the information period. The pSTS showed the same pattern at the choice period. These three regions are regarded as being important in social cognition and theory of mind, as they are often seen to be active in the process of perspective-taking (Gallagher and Frith, 2003; Saxe and Kanwisher, 2003; Frith and Frith, 2006; Vollm et al., 2006). Previous data substantiate that these regions are highly associated with dynamic belief updating, a critical process for aligning one's own and other's beliefs to predict correct outcomes (Baker et al., 2017; De Martino et al., 2017; Rusch et al., 2020). Taken together, it is possible that participants in our experiment were trying to scrutinize the product by dynamically referring to their own assessment of a product and by inferring to the reasoning behind a given rating. Oftentimes, when we shop online, we make our own opinion about a product based on the images and then analyze why other customers have given such rating. Therefore, activations in the mPFC and TPJ toward the rating condition at the information period may be a reflection of the process by which shoppers assess their own and others' opinions by reasoning about a given rating. Furthermore, the recruitment of the pSTS at the choice period indicates that when customer ratings are presented to shoppers, these social cognitive functions may continue from product evaluation to choice process.

Meanwhile, categorical analysis of our data demonstrated several neural areas showing the main effects of intention change and product information, but no interaction effect between the two factors was observed. The lack of interaction effect may be due to the combination of both increased and decreased purchase intentions to represent the changed condition. Nevertheless, the results accentuated the neural underpinnings of unchanged purchase intentions upon exposure to product information. In addition, subsequent parametric analysis effectively presented evidence of regions linearly modulated by the level of purchase intention change depending on the type of product information, such as the caudate in the price condition and the insula in the rating condition. Considering that the caudate engages in several functions, such as the adaptive decision-making, motivation, and emotion processing (Grahn et al., 2008; Stelzel et al., 2010; Doi et al., 2020), the inverse modulatory effect of the change-in-intention scores on caudate activity when price information is disclosed possibly conveys how consumers focus more on the negative side over the benefits of purchasing as their desires decrease. On the contrary, the modulation of insula activity by intention change based on customer ratings showed a negative relationship with left insula activity at the information period and a positive relationship with right insula activity at the choice period. Given that the insula incorporates various signals to determine the salience of stimuli as a prominent area in salience and emotion processing (Gogolla, 2017), this opposite relationship according to the period of decision-making may signify lateralization of insula activity, which leads to greater salience and emotion associated with rating information as the desires decrease or increase.

One of the strengths of this study is that the number of participants included in the analysis was 38, which was sufficient for an experimental neuroimaging study. The type of this study is an “experiment with increased behavioral realism,” in which consumer behavior is measured in a laboratory setting and follows a within-subject design (Viglia et al., 2021). Such design is considered especially advantageous in consumer research because data tend to contain less noise and have higher statistical power, and a smaller sample size is deemed adequate (Viglia et al., 2021). The sample size may be an important issue in neuromarketing research (Lim et al., 2019). In fact, the sample size was much smaller in fMRI neuroimaging studies than in other fields of study, mainly due to the financial burden of running studies, but showed acceptable levels of test-retest reliability (Bennett and Miller, 2010; Plichta et al., 2012). In a thorough evaluation of sample sizes of fMRI publications, the median sample size was 14.5, and high-impact neuroimaging journals in 2018 had a median sample size of 24 (Szucs and Ioannidis, 2020). Another strength lies in the way the stimulus is presented. The way one processes information is influenced by chronic disposition, which refers to one's stable orientation or motivation, and situational priming, which indicates the temporary impact of a particular scenario or situation (Lisjak et al., 2012). In order to reduce the cognitive load of stimulus processing and to induce heuristic processing to enable a more efficient design (Lim, 2015), the stimuli in this study consisted of pictorial illustrations with the exception of the response question shown to gauge one's willingness to purchase.

In addition to these strengths, several limitations should also be noted. First, as demographic factors such as age, gender, and income heavily influence consumer behavior (Kalyanam and Putler, 1997), including only young female adults as participants has the effect of excluding confounding factors but also limits the scope of interpretation. Moreover, information regarding income was not collected. Additionally, only lip-care products were used in the study, which poses a possible generalizability issue of the study. Future studies should incorporate a variety of product types such as utilitarian items and other hedonic social products that are readily used by male and female consumers of all ages.

## Conclusion

This study delineated the e-commerce consumer process and incorporated price and customer rating information to explore the neural substrates of changing purchase intentions for hedonic products. The results indicated that brain regions related to cognitive control and social cognition processing were involved differently depending on the type of information. In particular, the findings highlighted the employment of the FPC for an information-driven explorative decision-making strategy in changing purchase intentions during the evaluation phase. Furthermore, salience processing-related regions were importantly involved in maintaining purchase intentions despite referring to provided information during both evaluation and choice phases of the decision-making process. When information to help shoppers make a purchase decision was presented, social cognition-related regions were engaged in reference to customer ratings rather than price in both product evaluation and choice processes.

## Data Availability Statement

The original contributions presented in the study are included in the article/supplementary material, further inquiries can be directed to the corresponding author/s.

## Ethics Statement

The studies involving human participants were reviewed and approved by Institutional Review Board of Yonsei University Severance Hospital. The patients/participants provided their written informed consent to participate in this study.

## Author Contributions

HK and JK conceived and designed the experiments and performed the experiments. HK analyzed the data and drafted the manuscript. J-JK edited and revised the manuscript. All authors contributed to the article and approved the submitted version.

## Funding

This work was supported by the National Research Foundation of Korea (NRF) grant funded by the Korean government (MSIP) (No. NRF-2016R1A2A2A10921744).

## Conflict of Interest

The authors declare that the research was conducted in the absence of any commercial or financial relationships that could be construed as a potential conflict of interest.

## Publisher's Note

All claims expressed in this article are solely those of the authors and do not necessarily represent those of their affiliated organizations, or those of the publisher, the editors and the reviewers. Any product that may be evaluated in this article, or claim that may be made by its manufacturer, is not guaranteed or endorsed by the publisher.
